# Variations in micronutrient concentrations and retentions in *fufu* made from yellow-fleshed cassava as a function of genotype and processing methods

**DOI:** 10.3389/fnut.2024.1295609

**Published:** 2024-05-22

**Authors:** Martha Shirley Epiphaneia Williams-Ngegba, Oluseye Olusegun Onabanjo, Nyahabeh Mariama Anthony, Emmanuel Oladeji Alamu, Busie Maziya-Dixon, Emmanuel Babatunde Oguntona

**Affiliations:** ^1^Post-Harvest Engineering, Food and Nutrition Sciences, Sierra Leone Agricultural Research Institute, Freetown, Sierra Leone; ^2^Nutrition and Dietetics Department, Federal University of Agriculture, Abeokuta, Nigeria; ^3^Food and Nutrition Sciences Laboratory, International Institute of Tropical Agriculture (IITA), Ibadan, Nigeria

**Keywords:** β-carotene, genotype, retentions, yellow-fleshed, micronutrients, processing, *fufu*

## Abstract

**Introduction:**

The biofortification of staple foods such as cassava is one of the technological breakthroughs in the nutritional improvement of foods. *Fufu* is one of the fermented cassava products produced and consumed in major West African countries, including Sierra Leone, and the majority of the processes involved in its production have direct and indirect effects on its properties. This study looked at how the concentration and retention of micronutrients in yellow-fleshed cassava *fufu* varied depending on genotype and processing method.

**Methods:**

Six yellow-fleshed cassava root genotypes (TMS-070557, TMS-011371, TMS-011412, TMS-011663, TMS-083724, TMS-083774) and one white (TME 419 as a control) were processed into *fufu* using both conventional (oven and sun-dried) and traditional (bowl and river) methods. The Statistical Analysis System (SAS) version 9.4 was used to analyze data using means, percentages, analysis of variance and means separated by least significant differences (LSD).

**Results and Discussion:**

In the modified traditional river method, raw and cooked *fufu* samples had significantly higher β-carotene concentrations and true retention (TR) percentages (11.06  g/g (46.77%) and 4.54 g/g (16.94%), respectively) than other genotypes (p  <  0.0001). Modified traditional *fufu* processing methods increased total β-carotene concentrations, while raw roots showed a significant decrease in total carotenoid and β-carotene concentrations, regardless of genotype or processing method. Sun-drying was the most effective method, with significantly higher concentrations and TR percentages of iron (10.01  mg/kg, 18.02%) and zinc (11.49  mg/kg, 40.64%) in raw and cooked *fufu* samples. Genotype TMS-083724 outperformed both conventional *fufu* processing methods, displaying a significant total carotenoid concentration and true retention percentage. Finally, this study found that the concentrations and percentages of TR of micronutrients varied depending on the processing method and genotype. It is recommended that a modified traditional river *fufu* processing method be further developed and improved in order to maximize provitamin A carotenoids, concentrations, and percentage TR.

## 1 Introduction

Hidden hunger, also known as micronutrient deficiency, is a public health issue globally, especially among populations that rely on staple foods that are deficient in micronutrients, such as roots and tuber crops (1, 2). The geographical regions most affected by micronutrient deficiency are believed to be in the order of: South Asia (in particular, Bangladesh and India), Africa, and the Western Pacific in terms of severity. In Sierra Leone, 83% of preschool children and 60% of women of childbearing age are iron deficient (3), 17.2% of children 6–59 months suffer from Vitamin A deficiency, and anemia has been associated with Vitamin A in young children in the country (4). Deficiencies in the major micronutrients, vitamin A, iron, iodine, and zinc, are common among populations that consume plant-based diets (5). One of the technological breakthroughs in global nutritional improvement projects is the biofortification of staple foods such as cassava with micronutrients such as carotenoids, iron, and zinc (6, 7). Cassava is rich in carbohydrates but grossly deficient in other nutrients. It is majorly consumed in most African countries, including Sierra Leone, as a staple food. *Fufu* is one of the fermented cassava products that is produced and consumed in some West African countries like Nigeria, Ghana, Cameroon, and Sierra Leone (8–10).

In recent years, HarvestPlus has introduced some biofortified cassava genotypes into Sierra Leone through the International Institute of Tropical Agriculture (IITA). However, the concentrations and percentage of true retentions of micronutrients when these genotypes are subjected to different product processing methods are yet to be investigated. Reduction in the carotenoid contents during the processing of yellow-fleshed cassava root into some food forms has been reported (10–12). Most of the processes leading to the production of this food sometimes have direct and indirect influences on its properties. *Fufu* is traditionally produced into a wet paste or conventionally to obtain flour. *Fufu* may lose carotenoid, iron, zinc, and other micronutrients through leaching during processing, which could explain the differences in nutrient retention. Individuals processing cassava into *fufu* traditionally ferment roots in different handling materials such as bowls, plastic drums, and basins and use other natural resources like stagnant water or a flowing river. These have been noted to have implications on the nutrients and total quality of the products. Conventional *fufu* processing methods involve peeling, washing, size reduction, fermenting, decanting, pressing, drying, and milling the resulting *fufu* into flour. De Moura et al. (13) revealed that among several methods of processing, sun drying was more detrimental to the provitamin A levels (27–56% retention) in cassava than shade (59%) or oven drying (55–91%). Resource-poor producers mostly produce *fufu* and rely mainly on using low technologies to prepare *fufu*. It is, therefore, pertinent to determine the effects of some of these traditional methods on the availability of micronutrients in biofortified root samples and their percentage of true retention. Further, the complexity of modern cassava processing equipment and its high cost limits the local *fufu* producers to readily available domestic tools, some of which have been considered suitable to be used when processing yellow cassava owing to their ability to mitigate nutrient loss (10).

There is a dearth of information on the impact of traditional and conventional processing methods on nutrient concentrations and percentage true retentions of yellow-fleshed cassava genotypes grown in Sierra Leone. Hence, this study evaluated the variations of total carotenoids, β-carotene, iron, and zinc concentrations and percentage true retentions in yellow-fleshed cassava *fufu* as a function of processing methods and genotypes.

**Figure 1:**
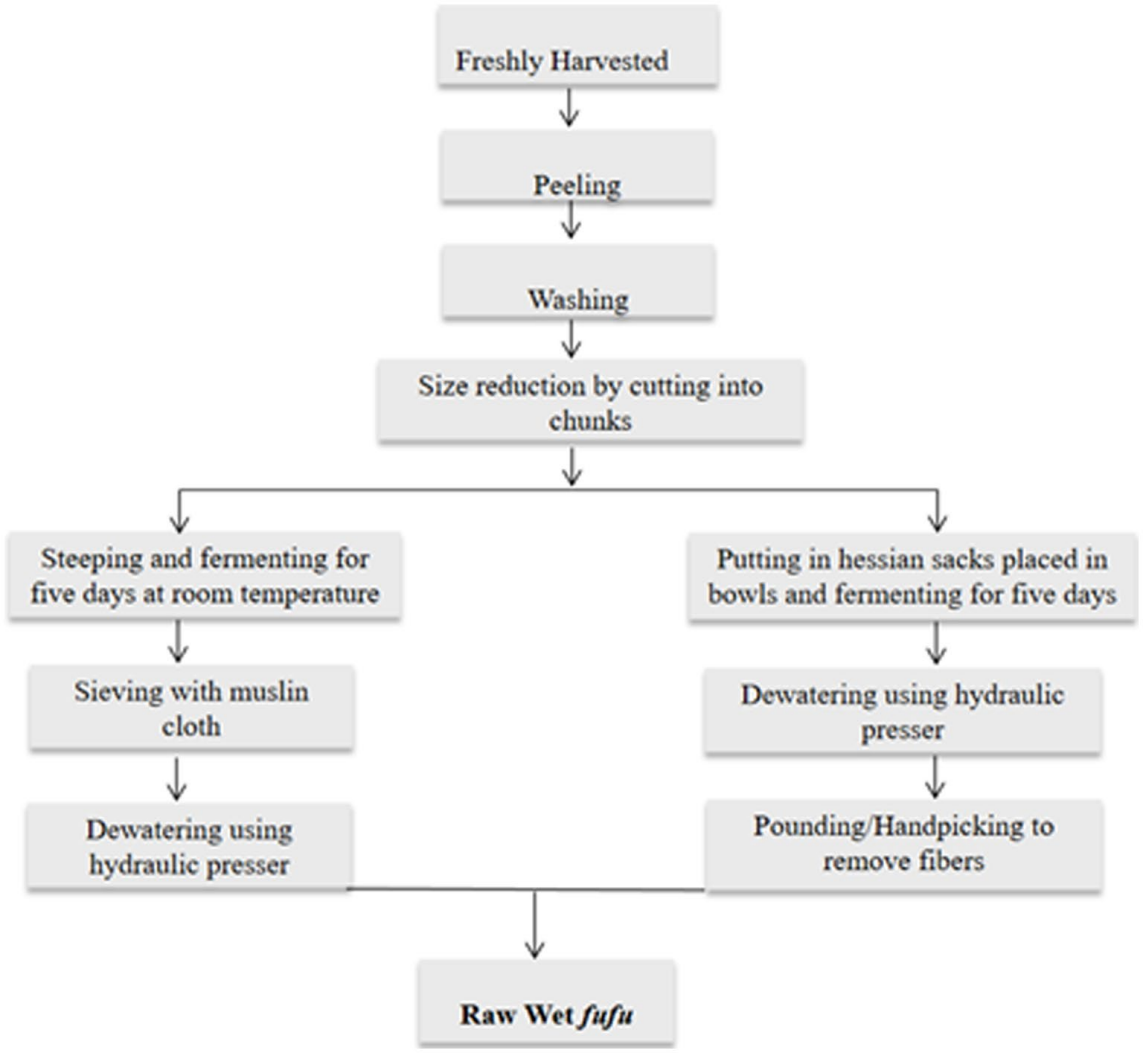
Flow chart of traditional raw *fufu* processing adapted from Maziya-Dixon et al. (14) and traditional river processing method from indigenes of Sierra Leone.

## 2 Materials and methods

### 2.1 Sources of raw materials

Six yellow-fleshed cassava (*Manihot esculenta*) improved genotypes and one white variety that were grown under rain-fed conditions in a randomized complete block design with two replications at the Njala Agricultural Research Centre (NARC) Experimental site in Kori Chiefdom, Moyamba District, Southern Region of Sierra Leone, West Africa were harvested at 14 months after planting. The genotypes were supplied by the International Institute of Tropical Agriculture (IITA) in Ibadan, Nigeria, to the Sierra Leone Agricultural Research Institute for adaptation study in Sierra Leone. Harvesting was done manually (early in the morning) by experienced personnel, and the storage roots were transported to the Dunsan Spencer processing unit at NARC.

### 2.2 Harvesting and preparation of cassava roots

Processing of the harvested cassava roots commenced within 60 min after harvesting. All sampling following harvesting was conducted in a dimly lit room to prevent oxidation in the shortest possible time. Three roots from each harvested genotypes were selected and washed thoroughly to remove dirt and other adhering particles. After which, stainless steel knives were used to cut the selected roots into four parts through longitudinal cuts from one extremity to its opposite, and four sections were obtained. Two opposite sections were discarded, and the remaining were peeled and washed in deionized water to prevent mineral contamination. The roots were then cut into tiny cubes and shared into two halves. The first half was wrapped in aluminium foil before being placed in sterilized Nasco whirl-pak bags for carotenoid and β-carotene analyses, while the remaining half was packed in the Sterilized whirl-paks for mineral analyses. All samples were kept in a −80^o^C freezer and were shipped to the International Institute of Tropical Agriculture (IITA) Ibadan, Nigeria, for carotenoids and mineral analyses of the raw cassava roots. The remaining roots were washed, peeled and processed into *fufu* using the traditional and conventional processing methods.

### 2.3 *Fufu* processing

#### 2.3.1 Traditional *fufu* processing

Traditional Bowl method: this is an improved method for *fufu* production in African as described by Maziya-Dixon et al. (14).

Modified traditional river method: this was done with slight modifications of the typical traditional *fufu* production methods by rural families in Sierra Leone. The term modified traditional river method belongs to a long-standing traditional *fufu* production in Sierra Leone, where, the cassava roots are peeled, washed, placed in sacks and left in a flowing river to ferment for days or weeks.

A quantity of 10 kg of freshly harvested cassava roots of each of the genotypes, were manually peeled using stainless steel knives. The peeled roots were then subjected to a rigorous washing process with deionized water in order to eliminate dirt and sand particles. After washing the roots were divided into two halves. One half was used for traditional bowl method whilst the other half was subjected to modified traditional river method.

For the traditional bowl method, 15–20 cm length pieces of the peeled and washed roots were steeped in water a plastic bowl for 5 days at ambient temperature with limited light. After 5 days, the fermented roots were passed through a plastic sieve to obtain a fine mash. The mash was then placed in a large plastic bowl and left to sediment for 24 h. The sediment (*fufu*) was then dehydrated with a muslin cloth and a IITA-manufactured hydraulic presser.

As for the modified traditional river, the roots were vertically cut opened, placed in hessian sacks, and steeped into large bowls of water for 5 days. After five days, the fermented roots were extracted, pulverized with a wooden mortar and pestle, and dehydrated using an IITA-manufactured hydraulic presser. The fibres in the resulting cake were handpicked and further passed through a plastic sieve to break up the cake.

The wet *fufu* from both methods were divided into two equal portions. A portion of the raw *fufu* was reserved for laboratory analysis and the remaining portion was cooked (see Figure 1).

#### 2.3.2 Processing of conventional (odorless) *fufu*

The process by which the odorless *fufu* was produced has been detailed by Omodamiro et al. (10).

A total of 10 kg of the harvested cassava roots of each genotype were manually peeled and washed using stainless steel knives. The peeled and washed roots were cut into 15–20 cm length pieces and steeped in water in a plastic bowl under dim light for 48 h at room temperature. The fermented roots were extracted from the plastic bowls after 24 h. The fibers were extracted by manually breaking the roots and passing them through a plastic sieve. Following 24 h of sedimentation, the water underwent additional decantation via a muslin cloth, hessian sack, and hydraulic presser (manufactured by IITA). Hand-masticating, weighing, and dividing the pressed cake in half for drying in the sun and the oven followed. One portion was dried for 48 h in plastic trays exposed to direct sunlight, while the other was dried for 72 h at 50°C in an oven. Using a 200 μm sieve and a stainless-steel grinder, the samples underwent an additional milling and sieving process to become fine flour. Samples were collected for laboratory analysis, and the remainder was utilized for cooking.

#### 2.3.3 Processing of cooked *fufu*

300 g sample of the wet fermented cassava paste and dried *fufu* flour were mixed with 300 mL and 400 mL of water, respectively, and cooked in a stainless-steel pot with continuous stirring with a wooden rod (15) for 20–25 min to obtain a sample of fermented cooked cassava dough (cooked *fufu*). The samples were divided into two, one was wrapped with aluminum foil and packaged into Nasco whirl pak (for total carotenoids and β-carotene analysis), whilst the other half was packaged into Nasco whirl pak only (for iron and zinc analysis) and stored at −80°C for further laboratory analyses.

### 2.4 Determination of provitamin A

#### 2.4.1 Total carotenoids and beta-carotene contents extraction of carotenoids

Carotenoid was extracted with petroleum ether and quantified using the spectrophotometric method described in the HarvestPlus Handbook for Carotenoid analysis (16). The method involves weighing 10 g of the homogeneous, representative of root and *fufu* samples, transferring them each into a mortar, adding 3 g of hyflosupercel (celite), and ground with 50 mL of cold acetone. The mortar, pestle, funnel, and residue were washed with small amounts of cold acetone, receiving the washings in the suction flask through the Buchner funnel using Whatman No 40-filter paper, and the extraction procedure repeated (until the residue was un-coloured), pooling all fractions together. 20 mL of Petroleum Ether (PE) was put into a 500 mL separatory funnel with a Teflon stop-cock. The samples were poured into a funnel and slowly washed with distilled water, allowing the water to flow along the funnel walls. The two phases were left to separate, and the lower aqueous phase was discarded. In order to remove all the acetone, washing was done thrice with distilled water (200 mL each time). The upper PE phase was collected in a 25 mL volumetric flask. The solution was passed through a small funnel containing 15 g of anhydrous sodium sulphate to remove residual water. The separation funnel was washed with PE, collecting the washings in the volumetric flask by passing through the funnel with sodium sulphate.

##### 2.4.1.1 Spectrometric readings and calculations

Samples were made up to volume with PE, and the absorbance was taken at 450 nm. The formula below was used to calculate the total carotenoids content (see Eq. 1).


(1) $$\mathrm{Total}\;\mathrm{carotenoids}\;\left(\mathrm{\mu g}/g\right)=\frac{A\times \mathrm{Volume}\;\left(\mathrm{mL}\right)\times 10\;4}{A1\%1\mathrm{cm}\times \mathrm{sample}\;\mathrm{weight}\;\left(g\right)}$$

Where: A = Absorbance; volume = total volume of extract (25 mL); A^1%^ 1 cm = Absorption coefficient of β-carotene in PE (2592) multiplied by 100 to give the carotenoid content in μg/100 g.

#### 2.4.2 β-carotene quantification

The organic phase used for spectrophotometric quantification of total carotenoid aliquots (15 mL) was transferred to a glass tube and dried by nitrogen evaporation (N-Evap 112, Organomation Associates, Berlin, MA, United States). The dry extract was dissolved in 2.0 mL of (1:1) methanol and methyl tert-butyl ether (MeOH): MTBE High-Performance Liquid Chromatography (HPLC)-grade after sonication (10 s) and agitation in a VWR multi-tube vortexer (2,400 rpm; 60 s) and filtered through a 0.22 μm poly-tetra-fluoro-ethylene (PTFE) filter immediately before injecting it into the same tube. Separation and quantification of carotenoids were achieved using a YMC Carotenoid S-5 C30 reversed-phase column (4.6 mm × 150 mm: particle size, 5 μm), with a YMC Carotenoid S-5 guard column (4.0 × 23 mm) in a HPLC system. Peaks were identified by comparing retention time and spectral characteristics against a pure standard from Carotene Nature GmbH, Lupsingen, Switzerland: β-carotene-N°0003 HPLC 96%. Duplicated injections of carotenoid standards at different concentrations through a linear regression using at least five-point (16 μg/mL, 32 μg/mL, 80 μg/mL, 160 μg/mL, and 240 μg/mL) analytical curves of (all-*E*)-lutein, α-carotene and (all-*trans*)-β-carotene—the relative response of the standards measured as mean peak areas were plotted against concentrations. The calibration curve was constructed using the least-squares linear regression methods. For the analytical curve, the linear regression was significant (*p* < 0.05) in the evaluated concentration ranges, which is also corroborated by the high values of the determination coefficient (*r*^2^ ≥ 0.9994) (16–18).

### 2.5 Determination of iron and zinc contents

Iron and zinc contents were determined using an Inductively Coupled Plasma Atomic Emission Spectrometer (ICP-AES) using methods described by Horwitz (19), Maziya-Dixon et al. (20), and Zarcinas et al. (21) for mineral profile analysis. Model Questron Technologies Corp. TL 6000. 0.5 g of sample was weighed into a 50 mL digestion tube, 2 mL of concentrated redistilled Nitric acid (HNO_3_), was added to each of the samples, left overnight for cold digestion, and placed on a digestion block starting with a temperature of 120°C. As the liquid dried off, 2 mL of concentrated HNO_3_ was further added. This step was repeated until the sample no longer gave off reddish–brown fumes or until the solution was clear. A solution of 50/50 (v/v) nitric acid and perchloric acid was then added, and the temperature was increased to 180°C–220°C, with the tap to wash the exhaust running. The samples were heated to dryness (leaving a white ash-like residue), removed from the digestion block, and allowed to cool to room temperature. The sample ash solutions were injected into the ICP-AES to determine the mineral content. All analyses were done in triplicate. The iron and zinc contents were calculated using the formula below (see Eq. 2).


(2) $$\begin{array}{l} \mathrm{Mineral}\;\mathrm{content}\left(\frac{\mathrm{mg}}{\mathrm{kg}}:\right)\;\\ \mathrm{Concentration}.\left(\mathrm{ppm}\right)X\;D.F./\mathrm{Sample}\;\mathrm{weight}\;\left(g\right)\\ D.F.,\;\mathrm{dilution}\;\mathrm{factor}\;=\;11\text{.} \end{array}$$

The percentage true retentions of the micronutrients were calculated using the formula below (see Eq. 3):


%TrueRetention:

(3) $$\frac{\begin{array}{l} \mathrm{Nutrient}\;\mathrm{per}\;\mathrm{gram}\;\mathrm{processed}\\ \times \mathrm{gram}\;\mathrm{of}\;\mathrm{food}\;\mathrm{after}\;\mathrm{processing} \end{array}}{\begin{array}{l} \mathrm{Nutrient}\;\mathrm{per}\;\mathrm{gram}\;\mathrm{of}\;\mathrm{raw}\;\mathrm{cassava}\;\mathrm{roots}\\ \times \mathrm{gram}\;\mathrm{of}\;\mathrm{raw}\;\mathrm{cassava}\;\mathrm{roots}\;\mathrm{before}\;\mathrm{processing} \end{array}}\times 100$$

### 2.6 Data analysis

Data analytical values are expressed in means. Two-way ANOVA was adopted for statistical analysis, followed by mean differences between treatment groups determined using LSD at a significance *p* <0.05. Data were analyzed using Generalized Linear Model (GLM) of Statistical Analysis System software (SAS, version 9.4). All total carotenoids and *β-carotene* were evaluated in replicates and iron and zinc were done in triplicate in the laboratory. All the reagents and chemicals used were laboratory grade.

## 3 Results

### 3.1 Mean squares from analysis of variance for total carotenoids, β-carotene, iron and zinc concentrations, and retentions in *fufu* as a function of genotype and processing methods

Table 1 presents the mean squares from the analysis of variance for the concentrations and the percentage true retentions of total carotenoids, β-carotene, iron and zinc in *fufu* produced from yellow-fleshed cassava genotypes using different processing methods. Significant variations (*p* < 0.001) were observed in the genotype, processing method and the interaction of processing method by genotype for all the micronutrients except for iron concentration in the raw and cooked *fufu* samples as well as the percentage true retention of the cooked samples that had no significant (*p* < 0.5) differences in the genotype and genotype by processing method interaction.

**Table 1:** Mean squares from analysis of variance for total carotenoids, **β**-carotene, iron and zinc concentrations and retentions in *fufu* as a function of genotype and processing methods.

Source	DF	Concentration raw *fufu*	%True retention of raw *fufu*	Concentration cooked *fufu*	% True retention of cooked *fufu*
Total Carotenoids					
Processing Method	3	183.910***	7253.606***	73.305***	591.023***
Genotype	6	216.299***	1345.504***	29.174***	288.697***
Processing Method*Genotype	18	11.911***	266.829***	4.204***	34.606***
β-carotene					
Processing Method	3	220.244***	7906.507***	54.765***	579.929***
Genotype	6	166.538***	1440.554***	17.441***	189.242***
Processing Method*Genotype	18	15.219***	329.170***	3.325***	49.796***
Iron					
Processing Method	3	23.786***	631.443***	95.308***	7630.728***
Genotype	6	10.494***	69.633***	17.830 ns	1153.473***
Processing Method*Genotype	18	3.826 ns	27.153***	15.222 ns	331.536 ns
Zinc					
Processing Method	3	26.548***	1244.376***	20.042***	494.925***
Genotype	6	11.602***	147.326***	9.363***	443.957***
Processing Method*Genotype	18	3.762***	108.010***	2.238***	120.717***

***Significant at *p* < 0.0001. ns, not significant.

### 3.2 Effect of processing methods and genotypes on micronutrient concentrations and percentage true retentions

#### 3.2.1 Total carotenoids

Table 2 shows that the total carotenoid concentration and percentage true retention of *fufu* samples varied significantly (*p* < 0.0001) across processing methods and genotypes. TMS 083724 and 083774 had significantly different total carotenoid concentrations (17.95 μg/g and 13.11 μg/g, respectively) in the traditional bowl method (*p* < 0.0001). The percentage of true retention varied from 34.63% (TMS 011663) to 68.02% (TMS-083724). Furthermore, cooking the raw *fufu* samples reduced total carotenoid concentrations and the percentage of true retentions across genotypes. TMS-083724 had the highest total carotenoid concentration and percentage true retention (8.20 μg/g and 22.58%) compared to other genotypes. Similarly, the modified traditional river method revealed the sequence of total carotenoid concentration and the corresponding percentage true retention in traditional bowl samples. TMS-083724 had the highest total carotenoid concentration (18.86 μg/g), followed by TMS-011371, which had significantly higher total carotenoid concentrations (*p* < 0.0001) than the other genotypes. Cooking the traditional river fermented wet pastes resulted in lower total carotenoid concentrations and percentage true retentions across all genotypes. TMS-083724 and TMS-011371 had the highest total carotenoid concentrations (8.99 μg/g and 7.95 μg/g, respectively), with true retention rates of 24.76 and 25.55%. TMS-083724 and TMS-011371 had significantly higher total carotenoids concentrations (14.08 μg/g and 13.37 μg/g, respectively) in the oven-dried *fufu* samples. However, when the raw *fufu* was cooked into a thick dough, there was a significant reduction in total carotenoid concentrations and corresponding percentage true retention. In sun-dried samples, TMS-083724 had a total carotenoid concentration of 8.67 μg/g and a true retention percentage of 17.19%. TMS-011371 had a total carotenoid concentration of 5.00 μg/g and a retention percentage of 10.63% compared to the other genotypes. The total carotenoid and true retention percentages were low across all genotypes in the cooked sun-dried *fufu* samples.

**Table 2:** Total carotenoids concentration (μg/g) and percentage true retention in *fufu* processed from traditional and conventional processing methods.

Treatment		Concentration (μg/g) in raw root	Concentration (μg/g) in raw *fufu*	% True retention (μg/g) of raw *fufu*	Concentration (μg/g) in cooked *fufu*	% True retention (μg/g) of cooked *fufu*
Processing Method	Genotype					
Traditional Bowl	Control	0.10^f^	0.01^g^	0.00^e^	0.00^f^	0.00^f^
	TMS 070557	8.1^c^	12.03^e^	44.76^c^	3.59^e^	11.42^c^
	TMS 011371	8.03^c^	15.77^b^	60.69^ab^	6.03^d^	19.09^b^
	TMS 011412	7.34^d^	12.83^d^	55.87^b^	5.19^c^	18.25^b^
	TMS 011663	3.65^e^	4.07^f^	34.63^d^	0.93^c^	6.39^e^
	TMS 083724	9.37^a^	17.95^a^	68.02^a^	8.20^a^	22.58^a^
	TMS 083774	8.85^b^	13.11^c^	41.58^cd^	4.99^b^	10.22^d^
Traditional River	Control	0.10^f^	0.01^f^	0.00^c^	0.00^g^	0.00^e^
	TMS 070557	8.10^c^	11.03^d^	31.98^d^	4.3^e^	13.7^d^
	TMS 011371	8.03^c^	18.85^a^	73.01^a^	7.95^b^	25.55^a^
	TMS 011412	7.34^d^	13.13^c^	67.7^a^	6.56^c^	23.08^b^
	TMS 011663	3.65^e^	5.07^e^	54.63^b^	2.45^f^	17.33^c^
	TMS 083724	9.37^a^	18.86^a^	66.64^a^	8.99^a^	24.76^a^
	TMS 083774	8.85^b^	14.55^b^	41.58^c^	5.87^d^	17.14^c^
Oven Dried	Control	0.10^f^	0.1^d^	0.00^g^	0.00^d^	0.00^f^
	TMS 070557	8.10^c^	6.42^c^	8.80^e^	0.99^d^	8.23^d^
	TMS 011371	8.03^c^	13.78^a^	29.15^a^	3.85^a^	15.64^b^
	TMS 011412	7.34^d^	3.76^c^	6.27^f^	0.25^d^	2.26^e^
	TMS 011663	3.65^e^	3.28^c^	12.59^c^	0.08^d^	1.52^e^
	TMS 083724	9.37^a^	14.08^a^	27.94^b^	3.35^b^	17.01^a^
	TMS 083774	8.85^b^	8.83^b^	11.75^d^	1.21^c^	9.28^c^
Sun Dried	Control	0.10^f^	0.00^e^	0.00^e^	0.00^c^	0.00^a^
	TMS 070557	8.10^c^	3.22^cd^	4.45^d^	0.05^c^	0.38^a^
	TMS 011371	8.03^c^	5.00^b^	10.63^b^	0.34^b^	2.82^a^
	TMS 011412	7.34^d^	3.07^d^	5.04^d^	0.18^bc^	1.69^a^
	TMS 011663	3.65^e^	2.29^d^	8.84^c^	0.07^c^	1.34^a^
	TMS 083724	9.37^a^	8.67^a^	17.19^a^	1.17^a^	8.47^a^
	TMS 083774	8.85^b^	4.14^bc^	5.21^d^	0.11^b^	0.84^a^

Values with different superscript letters in the same column within processing method are significantly different (*p* < 0.0001).

#### 3.2.2 β-carotene

Table 3 presents the concentrations (μg/g) and percentage true retention of β-carotene in *fufu* processed from seven genotypes and four processing methods. The total β-carotene concentrations increased in the processed raw *fufu* compared to the corresponding raw cassava roots across all the genotypes. For the traditional bowl method, TMS-083724 and TMS-083774 had significantly (*p* < 0.0001) higher total β-carotene concentrations (8.49 μg/g) compared to the other genotypes. A similar trend was observed in the percentage true retention, as TMS 083724 had the highest retention of 68.94%, followed by TMS 011371 with a 55% retention rate. Interestingly, this was not the case for TMS-083774, which had the lowest percentage true retention of 43.47%, suggesting that both genotype and processing method affect percentage true retention. When the raw *fufu* samples were cooked, reductions in total β-carotene concentration and corresponding true retention percentage across all the genotypes were observed. TMS-083724 had a higher total β-carotene concentration (5.99 μg/g) and true retention percentage (18.21%) than the other genotypes. However, in the modified traditional river method, TMS-011371 had the highest total β-carotene concentration of 18.56 μg/g compared to TMS-011663 (5.18 μg/g) with the lowest concentration. There was a significant difference (*p* < 0.0001) in the corresponding true retention percentage with TMS-083724 (77.80%) compared to the other genotypes. Significant differences (*p* < 0.0001) in the total β-carotene concentration and true retention percentage in the oven-dried *fufu* samples were observed. TMS-083724 and TMS-011371 had significantly (*p* < 0.0001) higher total β-carotene concentration and true retention percentages of (12.94 μg/g and 11.65 μg/g) and (28.34 and 26.53%) compared to other genotypes. A positive relationship was apparent between total β-carotene concentration and true retention in the samples regardless of the genotype, except for TMS-011663, which had a higher retention value of 8.21% and a mean concentration of 2.07 μg/g. A considerable downward trend was observed in total β-carotene concentration and corresponding percentage true retention when the raw *fufu* samples were further cooked. There was a substantial decrease in the mean total β-carotene concentration for the sun-dried samples, from 6.03 μg/g in the raw roots to 2.61 μg/g in the processed raw *fufu* samples. Cooking of the *fufu* samples drastically reduced the concentrations and true retention of β-carotene in all the genotypes.

**Table 3:** Total **β**-carotene concentration (μg/g) and percentage true retention of *fufu* processed from traditional and conventional processing methods.

Treatment		Concentration (μg/g) in raw root	Concentration (μg/g) in raw *fufu*	% True retention (μg/g) of in raw *fufu*	Concentration (μg/g) in cooked *fufu*	% True retention (μg/g) of cooked *fufu*
Processing Method	Genotype					
Traditional Bowl	Control	0.01^e^	0.01^e^	0.00^f^	0.00^c^	0.00^c^
	TMS 070557	7.53^b^	11.49^c^	43.56^d^	2.20^b^	7.55^b^
	TMS 011371	7.46^b^	16.16^a^	55.00^b^	4.54^a^	15.45^a^
	TMS 011412	6.72^c^	12.8^c^	51.23^c^	4.71^a^	18.09^a^
	TMS 011663	3.54^d^	4.17^d^	32.65^e^	0.02^c^	0.12^c^
	TMS 083724	8.49^a^	16.04^b^	68.94^a^	5.99^a^	18.21^a^
	TMS 083774	8.49^a^	12.03^c^	43.47^d^	4.74^a^	10.13^b^
Traditional River	Control	0.01^e^	0.10^e^	0.00^f^	0.00^e^	0.00^e^
	TMS 070557	7.53^b^	10.03^c^	32.68^e^	3.94^c^	13.5^d^
	TMS 011371	7.46^b^	18.56^a^	68.84^b^	5.91^ab^	20.45^b^
	TMS 011412	6.72^c^	15.51^b^	52.44^c^	7.02^a^	26.96^a^
	TMS 011663	3.54^d^	5.18^d^	51.79^c^	3.05^d^	21.54^b^
	TMS 083724	8.49^a^	14.8^b^	77.8^a^	7.29^a^	22.18^ab^
	TMS 083774	8.49^a^	13.23^b^	43.85^d^	4.59^bc^	13.91^c^
Oven Dried	Control	0.01^e^	0.10^f^	0.00^f^	0.00^b^	0.00^d^
	TMS 070557	7.53^b^	5.11^d^	7.54^d^	0.55^b^	4.94^bc^
	TMS 011371	7.46^b^	11.65^b^	26.53^b^	2.33^a^	12.07^a^
	TMS 011412	6.72^c^	2.46^e^	4.48^e^	0.10^b^	1.01^cd^
	TMS 011663	3.54^d^	2.07^e^	8.21^d^	0.09^b^	1.83^cd^
	TMS 083724	8.49^a^	12.94^a^	28.34^a^	2.59^a^	7.86^ab^
	TMS 083774	8.49^a^	7.14^c^	9.91^c^	1.38^ab^	11.00^a^
Sun Dried	Control	0.00^d^	0.00^d^	0.00^e^	0.00^a^	0.00^b^
	TMS 070557	1.96^c^	1.96^c^	2.91^d^	0.06^a^	0.49^b^
	TMS 011371	4.59^a^	4.59^a^	10.51^a^	0.36^a^	3.31^ab^
	TMS 011412	2.52^b^	2.52^b^	4.52^c^	0.25^a^	2.53^ab^
	TMS 011663	1.90^c^	1.90^c^	7.59^b^	0.03^a^	0.51^b^
	TMS 083724	4.99^a^	4.99^a^	10.93^a^	0.86^a^	6.87^a^
	TMS 083774	2.88^c^	2.88^c^	2.99^d^	0.03^a^	0.28^b^

Values with different superscript letters in the same column within processing method are significantly different (*p* < 0.0001).

#### 3.2.3 Iron

Table 4 depicts the iron concentration (mg/kg) and percentage true retention of *fufu* processed from traditional and conventional processing methods. It was observed that the concentration of iron in the raw *fufu* varied from 6.43 mg/kg to 10.90 mg/kg in the traditional bowl method. Iron retention in *fufu* samples also varied from 17.02% in the control sample to 29.51% in TMS-011412. Iron concentration and retention increased when the raw *fufu* samples were further cooked, and means ranged from 11.28 to 23.11% (5.33–8.54 mg/kg). The least retention was recorded in TMS-011663, and the highest retention was in Genotype TMS-011371. Notably, the modified traditional river method also observed a concentration and true retention percentage variation. The percentage retention of the raw *fufu* ranged from 22.61% (9.22 mg/kg) in TMS-083774 to 32.20% (10.52 mg/kg) in TMS-011663, respectively. Further, in the cooked samples of the method, the concentration and retention of iron varied from 17.22 to 26.51 (11.00–12.59%), while TMS-083774 had the highest retention values compared to TMS-083724 with the least retention values.

**Table 4:** Iron concentration (mg/kg) and percentage true retention of *fufu* processed from traditional and conventional processing methods.

Treatment		Concentration (mg/kg) in raw root	Concentration (mg/kg) in raw *fufu*	% True retention of raw *fufu*	Concentration (mg/kg) in cooked *fufu*	% True retention of cooked *fufu*
Processing Method	Genotype					
Traditional Bowl	Control	10.62^c^	10.41^ab^	17.02^b^	6.62^bc^	14.07^ab^
	TMS 070557	8.92^d^	6.43^b^	21.00^ab^	6.82^abc^	17.5^ab^
	TMS 011371	9.54^cd^	9.31^ab^	27.33^a^	8.54^ab^	23.11^a^
	TMS 011412	9.11^d^	9.18^ab^	29.51^a^	5.19^c^	14.75^ab^
	TMS 011663	12.20^b^	10.90^a^	26.20^ab^	5.33^c^	11.28^b^
	TMS 083724	16.49^a^	10.25^ab^	22.40^ab^	9.63^a^	15.07^ab^
	TMS 083774	10.87^bc^	8.16^ab^	22.04^ab^	6.93^abc^	16.46^ab^
Traditional River	Control	10.62^c^	8.60^ab^	23.76^a^	8.03^c^	19.57^a^
	TMS 070557	8.92^d^	9.71^ab^	25.95^a^	7.45^c^	21.43^a^
	TMS 011371	9.54^cd^	9.86^ab^	30.33^a^	9.31^bc^	25.19^a^
	TMS 011412	9.11^d^	7.93^b^	25.55^a^	7.11^c^	20.18^a^
	TMS 011663	12.20^b^	10.52^ab^	32.20^a^	11.47^ab^	24.28^a^
	TMS 083724	16.49^a^	12.35^a^	28.98^a^	11.00^ab^	17.22^a^
	TMS 083774	10.87^bc^	9.22^ab^	22.61^a^	12.59^a^	26.51^a^
Oven Dried	Control	10.62^c^	8.67^a^	16.16^a^	10.65^b^	38.38^a^
	TMS 070557	8.92^d^	7.62^a^	17.36^a^	9.96^bc^	42.48^a^
	TMS 011371	9.54^cd^	7.27^a^	15.06^a^	9.75^bc^	39.19^a^
	TMS 011412	9.11^d^	7.87^a^	18.74^a^	9.76^bc^	40.98^a^
	TMS 011663	12.20^b^	6.86^a^	13.16^a^	7.58^cd^	23.76^a^
	TMS 083724	16.49^a^	9.09^a^	11.76^a^	19.84^a^	46.02^a^
	TMS 083774	10.87^bc^	6.05^a^	11.75^a^	6.18^d^	21.76^a^
Sun Dried	Control	10.62^c^	9.73^a^	18.10^ab^	11.09^abc^	39.84^b^
	TMS 070557	8.92^d^	8.42^a^	18.43^ab^	9.74^c^	41.53^ab^
	TMS 011371	9.54^cd^	10.75^a^	19.26^a^	12.76^ab^	51.19^a^
	TMS 011412	9.11^d^	9.83^a^	22.06^a^	10.43^bc^	43.88^ab^
	TMS 011663	12.20^b^	12.58^a^	20.32^a^	13.55^a^	42.47^ab^
	TMS 083724	16.49^a^	9.84^a^	11.09^b^	12.42^abc^	28.82^c^
	TMS 083774	10.87^bc^	8.96^a^	16.88^ab^	10.44^bc^	36.76^bc^

Values with different superscript letters in the same column within processing method are significantly different (*p* < 0.0001).

The retention of iron in oven-dried *fufu* samples varied from 11.75 to 18.74% (6.05–7.87 mg/kg). TMS-011412 and TMS-083724 retained sizeable iron compared to TMS-083774, with this method’s lowest iron in the raw *fufu*. An increased iron concentration and retention were observed, ranging from 21.76 to 46.02% (6.18–19.84 mg/kg) in the cooked *fufu*. For sun-dried samples, the true retention percentage of the raw *fufu* ranged from 11.09% (9.84 mg/kg) in TMS-083724 to 22.06% (9.83 mg/kg) in TMS-011412. Cooking the raw *fufu* into thick dough also increased the concentration and retention of iron. The retention varied from 28.82 to 51.19% (12.42–12.76%). Again, TMS-0113717 had the highest concentration and retention values, while TMS-083724 had the lowest.

#### 3.2.4 Zinc

In Table 5, the traditional processing methods displayed considerable variations in zinc concentration and percentage true retention in the raw and cooked *fufu* samples across all the genotypes. For the traditional bowl method, TMS-070557 had a significantly (*p* < 0.0001) higher zinc concentration of 9.50 mg/kg, while TMS-011663 registered the lowest (5.03 mg/kg). The true retention percentage ranged from 12.14% in the control sample to 36.96% in TMS-070557. When the raw *fufu* samples were cooked, there was a decrease in zinc concentration and percentage true retention compared to the raw *fufu*. TMS-011371 had a higher zinc percentage true retention (23.11%), and TMS-011663 retained the least amount of zinc. The sequence of zinc concentration and the corresponding percentage true retention in traditional bowl samples were also observed in the modified traditional river method. TMS-083724 recorded the highest concentration and percentage true retention of 18.86 mg/kg (40.04%) compared to TMS-011412, with the least concentration and true retention percentage of 5.03 mg/kg (17.08%) in the raw *fufu* samples. The mean zinc true retention percentage was low across genotypes and processing methods in the cooked samples. TMS-011371 and TMS-01142 had the highest (33.11%) and least (14.79%) retained amounts of Zinc. It was evident that the zinc concentration in raw and cooked *fufu* dried in the oven significantly (*p* < 0.0001) varied across the genotypes.

**Table 5:** Zinc concentration (mg/kg) and percentage true retention of *fufu* processed from traditional and conventional methods.

Treatment		Concentration (mg/kg) in raw root	Concentration (mg/kg) in raw *fufu*	% True retention of raw *fufu*	Concentration (mg/kg) in cooked *fufu*	% True retention of cooked *fufu*
Processing Method	Genotype					
Traditional Bowl	Control	7.50^ab^	5.11^f^	12.14e	5.15^d^	14.07^bc^
	TMS 070557	9.37^a^	9.50^a^	29.75^b^	9.44^a^	17.50^b^
	TMS 011371	5.71^b^	7.54^b^	36.96^a^	7.62^b^	23.11^a^
	TMS 011412	8.63^a^	5.36^e^	18.24^cd^	4.84^d^	14.75^bc^
	TMS 011663	9.57^a^	5.07^f^	15.36^de^	4.94^d^	11.28^b^
	TMS 083724	9.38^a^	6.03^d^	23.12^c^	6.13^c^	15.07^bc^
	TMS 083774	9.14^a^	7.05^c^	22.66^c^	7.25^b^	16.46^bc^
Traditional River	Control	7.50^ab^	9.32^b^	37.50^bc^	9.12^b^	32.28^a^
	TMS 070557	9.37^a^	8.05^d^	20.64^c^	9.22^ab^	25.40^ab^
	TMS 011371	5.71^b^	6.71^e^	34.47^abc^	7.33^c^	33.11^a^
	TMS 011412	8.63^a^	5.03^g^	17.08^c^	4.94^e^	14.79^c^
	TMS 011663	9.57^a^	5.86^f^	22.90^c^	6.17^d^	16.69^bc^
	TMS 083724	9.38^a^	9.72^a^	40.04^a^	9.31^ab^	25.60^abc^
	TMS 083774	9.14^a^	8.91^c^	26.03^bc^	9.63^a^	23.80^abc^
Oven Dried	Control	7.50^ab^	4.89^c^	13.28^ab^	6.01^c^	31.66^a^
	TMS 070557	9.37^a^	6.87^a^	14.99^a^	7.30^a^	29.82^a^
	TMS 011371	5.71^b^	4.25^d^	14.73^a^	4.74^e^	31.74^a^
	TMS 011412	8.63^a^	4.97^c^	12.44^ab^	5.47^d^	24.26^a^
	TMS 011663	9.57^a^	3.92^e^	9.60^b^	4.44^f^	17.81^a^
	TMS 083724	9.38^a^	6.22^b^	14.18^ab^	6.78^b^	27.65^a^
	TMS 083774	9.14^a^	5.94^b^	13.74^ab^	6.44^c^	26.99^a^
Sun Dried	Control	7.50^ab^	4.96^d^	13.49^a^	6.15^cd^	32.35^a^
	TMS 070557	9.37^a^	6.30^a^	13.20^a^	7.24^a^	29.57^ab^
	TMS 011371	5.71^b^	4.03^e^	12.04^a^	4.78^e^	32.01^a^
	TMS 011412	8.63^a^	5.70^b^	13.52^a^	4.71^f^	20.9^b^
	TMS 011663	9.57^a^	5.14^cd^	10.60^a^	5.88^d^	23.55^ab^
	TMS 083724	9.38^a^	5.77^b^	11.42^a^	6.83^ab^	27.84^ab^
	TMS 083774	9.14^a^	5.49^bc^	12.32 ^a^	6.48^bc^	27.16^ab^

Values with different superscript letters in the same column within processing method are significantly different (*p* < 0.0001).

Further, the zinc concentration in the sun-dried samples varied from 4.03–6.30 mg/kg in the raw *fufu*, while the percentage of true retention of zinc in *fufu* samples also varied across the genotypes. For the cooked samples, the true retention percentage ranged from 20.90 to 32.35% (4.71–6.15 mg/kg). The least retention was observed in TMS-011412, while the highest was in the control sample. In the case of the sun-dried method, a variation in the concentration and true retention percentage were also observed. Zinc concentration decreased in the raw *fufu* compared to the roots for both methods. However, after cooking the raw roots, an increase in the zinc concentration and percentage true retention were also observed.

## 4 Discussion

Cassava roots are prone to physiological deterioration within 24 h after roots have been harvested and, therefore, need to be processed into different products shortly after harvesting. Consumption of cassava after the traditional preparation of dishes that require peeling, chopping, boiling, roasting, grating, and fermentation forms the dietary pattern of the Sierra Leonean population. Previous studies explored conventional (odorless) processing methods to produce *fufu* flour, most notably for convenience and storage stability (10, 22). *Fufu* is prepared by fermentation of cassava roots in water followed by sieving, dewatering, pressing, and cooking of the fermented paste or flour (10, 23). This study ascertained the retention of these nutrients during *fufu* processing using traditional and conventional processing methods. From the result, the significant variations observed in the genotype could be attributed to their genetic characterizations and/or corresponding processing methods. This finding is corroborated with previous studies by Maziya-Dixon et al. (20), Alamu et al. (24), and Eyinla et al. (25). Processing can potentially decrease the contents of carotenoids, iron, and zinc. Carotenoids are denatured by heat, light, oxygen, or a combination of all three (13, 26, 27). Increasing the surface area of cassava roots by fermentation, mashing, and oven/sun drying exposes carotenoids in the food matrix to increased light and oxygen, and cooking exposes the carotenoids to elevated temperatures (13, 26).

Processing often affects the amount of carotenoids in the consumed product. This study discovered increased β-carotene concentrations when traditional methods processed the raw storage roots into *fufu*. This increase in concentration might result from moisture and soluble solid losses, which were not accounted for, thereby increasing the concentrate and the total carotenoid and β-carotene levels per unit of *fufu*. Conversely, when the raw *fufu* was further cooked, a decrease was also observed in the concentrations that might have resulted from heat application during cooking. These findings agree with studies by Maziya-Dixon et al. (14), Maziya-Dixon et al. (15), Carvalho et al. (18), and Taleon et al. (28).

The food preparation process reduces the amount of nutrients in food, as such processes that expose foods to high levels of heat, light, and/or oxygen cause the most significant nutrient loss (29). However, nutrients can also be washed out of foods by fluids introduced during cooking. Different factors affect nutrient retention, including the type of food, cooking temperature, and time. The level of nutrients that can be retained and not lost by leaching with water is referred to as nutrient retention (30). In this study, it was noticed that the true retention percentage of total carotenoids and total β-carotene varied significantly among the different genotypes in *fufu* samples. It was viewed that an apparent relationship between total carotenoid and total β-carotene concentrations and their true retention percentage existed. These findings aligned with previous studies (12, 26).

Furthermore, using the oven resulted in a higher true retention percentage of total carotenoid and β-carotene. Sun drying is the most accessible and cheapest means of food preservation in Sierra Leone and other developing countries, but considerable losses of provitamin A occurred (21). Sun drying resulted in the highest reductions in total carotenoid and total β-carotene retentions compared to oven drying, which presumably might be due to continued enzymatic activity and exposure to air due to direct sunlight. The results are consistent with several prior studies (10, 18, 26, 31). Also, Fagbemi and Ijah (32) reported lower concentrations in sun-dried samples, which could be attributed to extended activities of bacteria due to delay in drying or the presence of some thermophilic microorganisms such as bacteria in the latter. The traditional river processing and fermentation method, which is the typical method used by rural Sierra Leoneans for *fufu* production, was modified during this study. The method was aptly mimicked, though the samples were fermented in bowls instead of placing them in hessian sacks inside the river or stream.

Interestingly, *fufu* obtained from this method had higher retention values compared to the traditional bowl and conventional *fufu* processing methods, which could be attributed to the large surface area of the roots and sacking of the chopped cassava chunk before fermentation thereby limiting oxidation, moisture loss, and weight changes. Iron and zinc retentions were comparably higher in *fufu* samples processed from conventional methods than in traditional processing methods with the mashing of fermented cassava chunks, sieving, sedimentation, and dewatering by decanting the cassava juice, thereby leaching mineral compounds into the decanted water. A similar observation was made by Maziya-Dixon et al. (15).

Furthermore, a different scenario was observed for the conventional *fufu* processing method, where there was a substantial decrease in total carotenoid and β-carotene concentrations in the raw *fufu* compared to fresh raw roots irrespective of genotype and drying method. However, in the oven-dried method, TMS-011371 and TMS-083724 (Table 3) recorded increased total carotenoid and β-carotene concentrations. Again, our results disclosed that the iron and zinc concentrations in the cooked *fufu*, regardless of genotype and processing methods, were significantly higher than those in the fresh roots and raw *fufu*, respectively. The mechanism for this increase is not fully understood as deionized water, a stainless still pot, and a wooden spoon were used to cook *fufu*. Our results corroborated with Maziya-Dixon et al. (15), who made a similar observation but attributed it to the water used to cook the *fufu*. A reverse observation was made when the fresh roots were processed into raw *fufu* among all the genotypes and processing methods; a reduction in iron and zinc concentrations was observed, possibly due to leaching out of the nutrients during the different processing stages to obtain the solid paste. Variations in the concentrations and percentage true retentions of the genotypes were observed, suggesting them as promising vehicles for micronutrient biofortification.

## 5 Conclusion

This study established that micronutrient concentrations and percentage true retention varied based on processing methods and genotypes. The total carotenoid and β-carotene concentrations increased when raw storage roots were processed into *fufu* using traditional methods, as opposed to the conventional *fufu* processing method, which showed a decrease in total carotenoid and β-carotene concentrations in raw *fufu* compared to fresh raw roots regardless of genotype or drying method. Again, our findings revealed that the iron and zinc concentrations in cooked *fufu*, regardless of genotype or processing method, were significantly higher than those in the raw roots and raw *fufu*. The modified traditional river method observed the highest true retention percentage of total β-carotene, while sun-drying was the best method for iron and zinc true retention percentages. Genotype TMS-083724 was superior by exhibiting sizeable total carotenoid concentration and true retention percentage in both conventional *fufu* processing methods.

## 6 Limitations and recommendations

This study evaluated the concentration and percentage true retention of micronutrients in biofortified genotypes introduced into Sierra Leone by IITA using different processing methods and at different stages. However, only six genotypes and four processing methods were evaluated due to time and budget constraints. Further studies should explore the concentrations and retentions at different processing stages to ascertain the critical concentration and percentage of true retention of these micronutrients. Hence, elucidation and improvement of the modified traditional river *fufu* processing method is recommended to maximize micronutrient concentrations and retentions.

## Data availability statement

The raw data supporting the conclusions of this article will be made available by the authors, without undue reservation.

## Author contributions

MW-N: Conceptualization, Data curation, Formal analysis, Investigation, Methodology, Resources, Validation, Visualization, Writing – original draft, Writing – review & editing. OO: Conceptualization, Data curation, Formal analysis, Investigation, Methodology, Resources, Supervision, Validation, Visualization, Writing – original draft, Writing – review & editing. NA: Conceptualization, Data curation, Formal analysis, Investigation, Methodology, Resources, Validation, Visualization, Writing – original draft, Writing – review & editing. EA: Conceptualization, Data curation, Formal analysis, Investigation, Methodology, Resources, Validation, Visualization, Writing – original draft, Writing – review & editing. BM-D: Conceptualization, Data curation, Formal analysis, Funding acquisition, Investigation, Methodology, Project administration, Resources, Supervision, Validation, Visualization, Writing – original draft, Writing – review & editing. EO: Conceptualization, Data curation, Formal analysis, Investigation, Methodology, Resources, Supervision, Validation, Visualization, Writing – original draft, Writing – review & editing.
