# Supplementary material for: Variations in micronutrient concentrations and retentions in fufu made from yellow-fleshed cassava as a function of genotype and processing methods
Source: Front Nutr. 2024 May 22;11:1295609. doi: 10.3389/fnut.2024.1295609 (PMC11152159; doi:10.3389/fnut.2024.1295609)
Supplement: Supplementary file 1 [file Table_1.pdf]

Supplementary Material

Variations in micronutrient concentrations and retentions in *fufu* made from yellow-fleshed cassava grown in Sierra Leone as a function of genotype and processing methods

Williams-Ngegba, Martha Shirley Epiphaneia.[1]*, Onabanjo, Oluseye Olusegun[2], Anthony, Nyahabeh Mariama[1] Alamu, Emmanuel Oladeji[1], Maziya-Dixon, Busie.[3]*, Oguntona, Emmanuel Babatunde[2]*

Correspondence: Corresponding Authors: Maziya-Dixon, B and Williams, M. S. E
B.Maziya-Dixon@cgiar.org; mujearose@gmail.com

Supplementary Table 1: List of yellow-fleshed cassava roots harvested and screened

No.	Genotype	No.	Genotype
1.	IITA – TMS – IBA061635	11.	IITA – TMS – IBA011663
2.	IITA – TMS – IBA011206	12.	IITA – TMS – IBA011181
3.	IITA – TMS – IBA011404	13.	IITA – TMS – IBA011172
4.	IITA – TMS – IBA950379	14.	IITA – TMS – IBA090581
5.	IITA – TMS – IBA083724	15.	IITA – TMS – IBA070520
6.	IITA – TMS – IBA070557	16.	IITA – TMS – IBA070593
7.	IITA – TMS – IBA011371	17.	IITA – TMS – IBA083774
8.	IITA – TMS – IBA011368	18.	IITA – TMS – IBA085394
9.	IITA – TMS – IBA070738	19.	IITA – TMS – IBA070749
10.	IITA – TMS – IBA011412	20.	IITA – TMS – IBA010040
		21	TME 419
